# Low use of statins for secondary prevention in primary care: a survey in a northern Swedish population

**DOI:** 10.1186/s12875-016-0505-0

**Published:** 2016-08-11

**Authors:** Gunnar Nilsson, Eva Samuelsson, Lars Söderström, Thomas Mooe

**Affiliations:** 1 Department of Public Health and Clinical Medicine, Unit of Research, Education and Development - Östersund, Umeå University, Umeå, Sweden; 2 Department of Public Health and Clinical Medicine, Umeå University, Umeå, Sweden; 3 Unit of Research, Education and Development, Östersund Hospital, Region Jämtland, Härjedalen Sweden

**Keywords:** Cardiovascular disease, Statins, Myocardial infarction, Secondary prevention

## Abstract

**Background:**

Cholesterol-lowering therapy with statins is recommended in established cardiovascular disease (CVD) and should be considered for patients at high cardiovascular risk. We surveyed statin treatment before first-time myocardial infarction in clinical practice compared to current guidelines, in patients with and without known CVD in primary care clinics with general practitioners (GPs) on short-term contracts vs. permanent staff GPs.

**Methods:**

A total of 931 patients (345 women) in northern Sweden were enrolled in the study between November 2009 and December 2014 and stratified by prior CVD, comprising angina pectoris, revascularisation, ischaemic stroke or transitory ischaemic attack, or peripheral artery disease. Primary care clinics were classified by the proportion of GP salaries that were paid to GPs working on short-term contracts: low (0–9 %), medium (10–39 %), or high (≥40 %). We used logistic regression to identify determinants of statin treatment.

**Results:**

Among patients with prior CVD, only 34.5 % received statin treatment before myocardial infarction. The probability of statin treatment decreased with age (≥70 years OR 0.30; 95 % CI 0.13–0.66) and female gender (OR 0.39; 95 % CI 0.20–0.78) but increased in patients with diabetes (OR 3.52; 95 % CI 1.75–7.08). Among patients with prior CVD, the type of primary care clinic was not predictive of statin treatment. In the entire study cohort, 17.3 % of patients were treated with statins; women < 70 years old were more likely to receive statin treatment than women ≥70 years old (OR 3.24; 95 % CI 1.64–6.38), and men ≥70 years old were twice as likely to be treated with statins than women of the same age (OR 2.22; 95 % CI 1.31–3.76) after adjusting for diabetes and CVD. Overall, patients from clinics with predominantly permanent staff GPs received statin therapy less frequently than those with GPs on short-term contracts.

**Conclusions:**

In patients with prior CVD we found considerable under-treatment with statins, especially among women and the elderly. Methodologies for case findings, recall, and follow-up need to be improved and implemented to reach the goals for CVD prevention in clinical practice.

**Electronic supplementary material:**

The online version of this article (doi:10.1186/s12875-016-0505-0) contains supplementary material, which is available to authorized users.

## Background

Statin treatment reduces cardiovascular (CV) morbidity and mortality in patients at increased risk of CV events [1–5]. The Scandinavian simvastatin survival study group (4S) was the first to report decreased CV mortality from statin treatment [5]. Several different statins have since become available at low cost as generic drugs. The pharmacological mechanism common to all statins is inhibition of the rate controlling enzyme Hydroxymethylglutaryl-CoA reductase in cholesterol synthesis [6], whereas the relative efficiency depends on the dose and type of statin [7]. The most commonly reported adverse effects related to statins are muscle symptoms, and asymptomatic liver enzyme elevation [8–12]. The risk of incident diabetes is slightly increased by statins, but it is outweighed by the total CV risk reduction in treated patients [9, 13, 14].

Treatment of patients with previous cardiovascular disease (CVD) (i.e., secondary prevention) targets patients at very high CV risk, in contrast to treatment of persons apparently free from disease (i.e., primary prevention) [15]. Other patients with a very high or high total CV risk are those with diabetes (type 2 diabetes or type 1 diabetics with microalbuminuria), chronic kidney disease, or very high levels of individual risk factors [15, 16]. Statin treatment should be offered to women with the same therapeutic targets as men [1, 15, 17, 18]. Previous trials have had positive results with statin therapy among elderly patients [1, 19, 20]. In patients with established CVD, and there is evidence of the same relative risk reduction up to 75–80 years of age [21, 22].

A scoring algorithm can be used to estimate CV risk in patients without previously diagnosed CVD, e.g. The Swedish SCORE chart for cardiovascular risk (10-year risk of CV death is calculated from age, sex, smoking status, systolic blood pressure, and total serum cholesterol) [23], and several algorithms have been put forth [24–27]. The restriction of SCORE to ages 40–65 years is a problem because patients over 65 years of age are also eligible for preventive drug treatment. A SCORE value ≥5 % is proposed to be the cut-off for defining patients at high CV risk who could benefit from lipid-lowering drug treatment [28, 29].

To identify patients at increased risk of CVD, the participation of general practitioners (GPs) is crucial [29], but the implementation of treatment guidelines in practice may still be insufficient. Inadequate knowledge, time constraints, and insufficient patient compliance are barriers to implementing guidelines on CVD prevention [30–32]. Concerns have also been raised regarding overestimating risk and the consequences of overusing pharmacotherapy in national populations [33–35].

Lower adherence to therapy is associated with the patient’s understanding of risk in relation to disease [36], the provider-patient relationship, and continuity of care [37]. However, before barriers to implementation of risk-adjusted prevention in primary care can be dealt with, they must be identified [34, 35, 38–40]. What still remains to bridge, is the gap between clinical practice and the optimal treatment with statins according to scientific evidence. Therefore, we designed a population-based survey of patients hospitalised with first-time myocardial infarction (MI). The primary aim of this study was to assess treatment with statins prior to first-time MI, in patients with and without previously diagnosed CVD. Another aim was to assess treatment differences related to primary care clinics’ use of GPs on short- term contracts.

## Methods

### Setting and participants

We conducted a population-based survey in the region of Jämtland Härjedalen, northern Sweden, which has one central cardiology unit and 21 primary care clinics run by the regional health authorities. Roughly half of the patients live in rural communities, with primary care clinics available in each. Participating patients were hospitalised with first-time MI type 1 according to the universal definition [41, 42] between November 26, 2009, and December 31, 2014. The patients were identified from a population-based secondary prevention study after acute coronary syndrome (ACS), “The Nurse-Based Age Independent Intervention to Limit Evolution of Disease After Acute Coronary Syndrome (NAILED ACS) Risk Factor Trial”; the outline of this study was published previously [43]. Baseline medical data and demographics were recorded during the initial hospitalisation by experienced nursing staff. The patients were stratified by prior CVD, which comprised angina pectoris, revascularisation (coronary artery bypass grafting or percutaneous coronary intervention), ischaemic stroke or transitory ischaemic attack (TIA), or peripheral artery disease (PAD). PAD comprised leg artery disease, a stenosing lesion of the carotid, or atherosclerotic aneurysm. The 10-year risk of fatal CV events was manually estimated from the pre-existing baseline data for patients 40–65 years of age at study entry without prior CVD or diabetes, according to the Swedish SCORE chart in use during the study period [23]. The primary care clinics within the study area were classified according to the percent of salaries allocated to GPs working on short-term contracts vs. salaries paid to GPs on the permanent staff between 2010 and 2014. This data was provided by the regional health authorities. 

### Statistical analysis

Patient characteristics are presented as proportions or means. We used the chi-squared test or Fisher’s exact test, as appropriate, to compare proportions and the two-sided Student’s *t*-test to compare means. In a trend analysis, age was stratified in 10-year intervals. In the regression model, age was dichotomised at 70 years, the mean age of the study population. Primary care clinics were stratified into three levels according to the primary care clinics’ use of GPs on short-term contracts, with approximately one-third of study patients in each level: low (0–9 %), medium (10–39 %), and high (≥40 %) short-term clinics. We used univariate logistic regression with patients stratified by prior CVD to identify patient determinants that were predictive of statin treatment. In a multivariable logistic model, we included the interaction term “age ≥70 years × female gender” with age ≥70 years, female gender, diabetes, and prior CVD as covariates. The SCORE variable was not included in the regression model due to bidirectional causality between statin treatment and the subsequent SCORE value. In an interaction model of age and gender, we calculated ORs for statin treatment adjusted for diabetes and prior CVD. The level of significance was set at *p* < 0.05. Sample size calculations were performed using WINPEPI, version 11.54 [44], and all other statistical analyses were performed in IBM SPSS version 22.

## Results

### Descriptive data

The study included 931 patients (345 women) with a first-time MI. The mean patient age was 74.7 years in women and 68.2 years in men. Most study patients (60.5 %) were current or previous manual workers. A complete background of the study population was described elsewhere [43].

Among the complete study sample, 4.1 % reported prior revascularisation and 5.6 % previous ischaemic stroke or TIA. Current diagnoses of angina pectoris, hypertension, diabetes, and PAD were reported by 11.7 %, 54.9 %, 18.5 %, and 2 % of study patients, respectively (Table 1). A total of 17.3 % of patients were being treated with statins at the time of admission to the hospital. Prior CVD was diagnosed in 166 of 931 patients (17.8 %) (Table 1). Patients with prior CVD were older, less often current smokers, more often diagnosed with diabetes, and received CV drugs more frequently. The low density lipoprotein (LDL) cholesterol levels were 3.0 (1.1 SD) and 3.3 (1.1 SD) mmol/L in patients with and without prior CVD, respectively (*p* = 0.010; Table 1).

**Table 1:** Characteristics of patients admitted for first-time myocardial infarction

Characteristic	Total *n* = 931	Prior CVD *n* = 166 (17.8 %)	No prior CVD *n* = 765 (82.2 %)	*p*
Age at admission to hospital, mean (SD)	70.6 (12.2)	78.3 (9.4)	68.9 (12.1)	<0.001
Female gender	345 (37.1 %)	71/166 (42.8 %)	274/765 (35.8 %)	0.093
Manual worker	531 (60.5 %)	96/151 (63.6 %)	435/727 (59.8 %)	0.392
Current smoker	198 (21.7 %)	21/159 (13.2 %)	177/755 (23.4 %)	0.004
Systolic blood pressure, mmHg, mean (SD) (67 missing)^a^	142 (18.4)	143 (18.7)	142 (18.3)	0.517
Diastolic blood pressure, mmHg, mean (SD) (67 missing)^a^	81 (11.2)	78 (12.5)	82 (10.7)	<0.001
Total cholesterol, mmol/L, mean (SD) (44 missing)	5.2 (2.2)	4.9 (1.3)	5.3 (2.4)	0.064
LDL cholesterol, mmol/L, mean (SD) (56 missing)	3.2 (1.1)	3.0 (1.1)	3.3 (1.1)	0.010
ST-elevation myocardial infarction	321 (34.5 %)	36/166 (21.7 %)	285/765 (37.3 %)	<0.001
Past medical history				
Revascularisation (CABG/PCI)	38 (4.1 %)	38/166 (22.9 %)	NA	-
Ischaemic stroke or TIA	52 (5.6 %)	52/166 (31.3 %)	NA	-
Present conditions				
Angina pectoris, current diagnosis	109 (11.7 %)	109/166 (65.7 %)	NA	-
Peripheral artery disease (PAD)	19 (2.0 %)	19/166 (11.4 %)	NA	-
Hypertension, current diagnosis	511 (54.9 %)	124/166 (74.7 %)	387/765 (50.6 %)	<0.001
Diabetes, current diagnosis	172 (18.5 %)	49/166 (29.5 %)	123/765 (16.1 %)	<0.001
Medication at admission to hospital				
ACE inhibitor/ARB	292 (31.5 %)	69/165 (41.8 %)	223/763 (29.2 %)	<0.002
Beta-blockers	265 (28.6 %)	89/165 (53.9 %)	176/762 (23.1 %)	<0.001
Calcium channel blockers	207 (22.4 %)	60/164 (36.6 %)	147/762 (19.3 %)	<0.001
Diuretics	248 (26.8 %)	72/165 (43.6 %)	176/762 (23.1 %)	<0.001
Long-acting nitroglycerine	49 (5.3 %)	45/165 (27.3 %)	4/763 (0.5 %)	<0.001
Statins	161 (17.3 %)	57/165 (34.5 %)	104/763 (13.6 %)	<0.001
Acetylsalicylic acid	222 (23.9 %)	112/165 (67.9 %)	110/763 (14.4 %)	<0.001

*ACE* angiotensin-converting-enzyme inhibitor, *ARB* angiotensin-receptor blocker, *CABG* coronary artery bypass grafting, *CVD* cardiovascular disease, a composite of diagnoses including angina pectoris, prior revascularisation, ischaemic stroke/TIA, or PAD (leg artery disease, a stenosing lesion of the carotid or atherosclerotic aneurysm), Current diagnosis of diabetes is type 1 or type 2. *LDL* low density lipoprotein, *PCI* percutaneous coronary intervention, *TIA* transitory ischaemic attack, *NA* not applicable

^a^Last representative blood pressure before admission to hospital

Simvastatin was the most prescribed statin, with 139 of the 161 patients on statins (86.3 %) receiving it, at a median dose of 20 mg in men and women. Twenty patients received atorvastatin. Only nine patients were treated with other statins or lipid-lowering drugs (bezafibrat, ezetimib) as single or combination treatment. High statin doses were uncommon; 6/161 patients were treated with 80 mg of simvastatin or atorvastatin. Sixty-two percent of the study patients were registered to a primary care clinic where ≥10 % of GPs served on short-term contracts (Table 2).

**Table 2:** Primary care clinics by proportion of GP salaries paid to GPs working on short-term contracts

Proportion of GP salaries paid to GPs working on short-term contracts	Number of primary care clinics	Number of patients (%)
Low (0–9 %)	11	353 (38 %)
Medium (10–39 %)	6	333 (36 %)
High (≥40 %)	4	245 (26 %)
Total	21	931 (100 %)

### Key findings

Among patients with prior CVD, 34.5 % were on statin treatment before admission for MI compared to 13.6 % of patients with no prior CVD (*p* < 0.001). Diabetes patients were treated with statins more often than non-diabetics. Within the entire study cohort, patients from clinics with predominantly permanent staff GPs received statin therapy less frequently than those with GPs on short-term contracts. Among patients with a risk SCORE value ≥5 % (age 40–65 years without prior CVD or diabetes), only 4.3 % (3/70) received statin treatment, compared to 7.6 % (11/144) of patients with a risk SCORE value <5 % (Table 3).

**Table 3:** Statin treatment prior to first-time myocardial infarction according to patient and primary care clinic characteristics (*n* = 928)

Characteristics	Treatment with statins	*p*
Patient characteristics		
Female gender	57/344 (16.6 %)	0.630
Male gender	104/584 (17.8 %)	
Age <70 years	67/433 (15.5 %)	0.158
Age ≥70 years	94/495 (19.0 %)	
Manual workers	95/528 (18.0 %)	0.626
Non-manual employees and self-employed	58/347 (16.7 %)	
Prior cardiovascular disease (CVD)	57/165 (34.5 %)	<0.001
No prior CVD	104/763 (13.6 %)	
Diabetes, current diagnosis	84/171 (49.1 %)	<0.001
Diabetes not recorded	77/757 (10.2 %)	
SCORE <5 %	11/144 (7.6 %)	0.728
SCORE ≥5 % - <10 %	3/55 (5.5 %)	
SCORE ≥10 %	0/15 (0.0 %)	
Proportion of GP salaries paid to GPs working on short-term contracts		
0–9 % (low short-term clinics)	48/351 (13.7 %)	0.047
10–39 % (medium)	54/255 (21.2 %)	
≥ 40 % (high)	59/322 (18.3 %)	

CVD is a composite of diagnoses including angina pectoris, prior revascularisation, ischaemic stroke/transitory ischaemic attack, or peripheral artery disease (PAD). PAD comprised leg artery disease, a stenosing lesion of the carotid or atherosclerotic aneurysm. Current diagnosis of diabetes includes diabetes of type 1 or type 2. SCORE: Systematic Coronary Risk Estimation, assessed in patients aged 40–65 years without prior CVD or diabetes. Three cases were missing data on statins and 53 on employment status

### Patients with prior CVD

In prior CVD patients, treatment with statins decreased with each 10-years age interval (*p* < 0.001 for trend; Table 4, and Additional file 1). Decreasing statin treatment with higher age was also observed after stratification according to the type of primary care clinic: low short-term (*p* = 0.028 for trend); medium to high short-term (*p* = 0.002 for trend). Crude ORs for statin treatment in patients ≥70 years and female patients were both inversely correlated with statin treatment, and the probability of statin treatment was not significantly different between types of primary care clinics (Table 5). In contrast to statins, treatment with other CV drugs, such as angiotensin-converting enzyme (ACE) inhibitors, angiotensin receptor blockers (ARBs), beta-blockers, calcium channel blockers, and acetylsalicylic acid, did not decline with age.

**Table 4:** Statin treatment prior to first-time myocardial infarction according to age (*n* = 928)

Age at hospital admission, years	Prior CVD (*n* = 165)	No prior CVD (*n* = 763)
≤49	1/1 (100 %)	4/45 (8.9 %)
50–59	2/4 (50 %)	13/117 (11.1 %)
60–69	15/26 (57.7 %)	32/240 (13.3 %)
70–79	20/53 (37.7 %)	36/204 (17.6 %)
80–89	18/66 (27.3 %)	17/126 (13.5 %)
≥90	1/15 (6.7 %)	2/31 (6.5 %)
Total	57/165 (34.5 %)	104/763 (13.6 %)

*CVD* cardiovascular disease, a composite of diagnoses including angina pectoris, prior revascularisation, ischaemic stroke/transitory ischaemic attack, or peripheral artery disease (PAD). PAD comprised leg artery disease, a stenosing lesion of the carotid, or atherosclerotic aneurysm. Prior CVD, *p* < 0.001 for trend. No prior CVD, *p* = 0.438 for trend

**Table 5:** Crude odds ratios (ORs) for statin treatment before first-time myocardial infarction stratified by cardiovascular disease (CVD) (*n* = 928)

Characteristics	Prior CVD *n* = 165		No prior CVD *n* = 763	
Patient characteristics	OR (95 % CI)	p	OR (95 % CI)	p
Age ≥70 years at admission to hospital	0.30 (0.13–0.66)	0.003	1.30 (0.86–1.96)	0.222
Female gender	0.39 (0.20–0.78)	0.008	1.19 (0.78–1.82)	0.422
Diabetes	3.52 (1.75–7.08)	<0.001	11.08 (6.97–17.62)	<0.001
Proportion of GP salaries paid to GPs working on short-term contracts				
0–9 % short-term	Reference		Reference	
10–39 % short-term vs. 0–9 %	1.38 (0.61–3.14)	0.437	1.79 (1.07–2.98)	0.026
≥ 40 % to short-term vs. 0–9 %	2.02 (0.92–4.40)	0.078	1.23 (0.74–2.05)	0.425

CVD is a composite of diagnoses including angina pectoris, prior revascularisation, ischaemic stroke/transitory ischaemic attack, or peripheral artery disease (PAD). PAD comprised leg artery disease, a stenosing lesion of the carotid, or atherosclerotic aneurysm. Current diagnosis of diabetes is type 1 or type 2

### Analyses including the entire patient cohort: Statin treatment by gender and age

The adjusted logistic model revealed a strong interaction between age and female gender with adjusted ORs for statin treatment (95 % CI) of 1.29 (0.79–2.11) for age ≥70 years; 1.88 (1.02–3.50) for female gender; and 0.24 (0.11–0.54) for age ≥70 × female gender, with positive ORs for diabetes and prior CVD (Table 6). Among women, the probability of statin treatment was approximately three-times as high in women <70 years of age compared to women ≥70 years of age. In patients ≥70 years of age, the probability of statin treatment in men was approximately twice as high as in women after adjusting for prior CVD and diabetes (female patients ≥70 years old served as a reference; Table 7).

**Table 6:** Adjusted odds ratios (ORs) for statin treatment prior to first-time myocardial infarction (*n* = 928)

Characteristic	Adjusted OR (95 % CI)	*p*
Age ≥70 years	1.29 (0.79–2.11)	0.305
Female gender	1.88 (1.02–3.50)	0.044
Age ≥70 years × female gender	0.24 (0.11–0.54)	<0.001
Diabetes	8.55 (5.72–12.79)	0.001
Prior cardiovascular disease (CVD)	3.27 (2.08–5.13)	<0.001

CVD is a composite of diagnoses: including angina pectoris, prior revascularisation, ischaemic stroke/transitory ischaemic attack, or peripheral artery disease (PAD). PAD comprised leg artery disease, a stenosing lesion of the carotid, or atherosclerotic aneurysm. Current diagnosis of diabetes is type 1 or type 2

**Table 7:** Adjusted odds ratios (ORs) for statin treatment by age and gender (*n* = 928)

Gender	Age at admission for first-time myocardial infarction	
	<70 years OR (95 % CI)	≥70 years OR (95 % CI)
Male	1.72 (0.99–2.99) (*n* = 326)	2.22 (1.31–3.76) (*n* = 258)
Female	3.24 (1.64–6.38) (*n* = 107)	1.00 (*n* = 237)

Females ≥70 years of age served as the reference. ORs were adjusted for prior cardiovascular disease (CVD) and diabetes. CVD is a composite of diagnoses: including angina pectoris, prior revascularisation, ischaemic stroke/transitory ischaemic attack, or peripheral artery disease (PAD). PAD comprised leg artery disease, a stenosing lesion of the carotid, or atherosclerotic aneurysm. Diabetes includes type 1 and type 2

## Discussion

Only one-third of patients with a first-time MI and previously diagnosed CVD were being treated with statins before the MI, despite guidelines that support active treatment of patients with known CVD [15, 29, 45]. In prior CVD patients, increasing age and female gender were associated with a lower probability of statin treatment, whereas diabetes was associated with a higher probability of statin treatment.

Our results revealed barriers to statin treatment for secondary prevention. The mean LDL cholesterol levels (3.0 mmol/L) exceeded the target level for high-risk patients according to the former and present European guidelines for CVD prevention in clinical practice [29, 46]. The Swedish guidelines for treatment of CVD during the study period recommended a target LDL cholesterol level <2.5 mmol/L in patients at very high CV risk [47].

Reasons for the underuse of statin treatment may be several: a lack of secondary prevention programmes; patients’ diminishing adherence to therapy over time; experienced or expected side effects of statins; and insufficient adherence to clinical guidelines among physicians.

The quality of CV risk management is associated with routines for case finding, patient follow-up, and CVD risk assessment integrated into the electronic medical record system [48]. Follow-up programmes delivered in primary care or as a nurse-based telephone follow-up have shown promising results with improved CV risk factors and treatment for coronary disease [43, 49–51].

Adherence to medication is associated with the access to and continuity of healthcare [37]. Within our study area, the continuity of primary care was impaired due to lack of GPs working as permanent staff. However, fewer patients received statin treatment at primary care clinics with predominantly permanent staff GPs, for unknown reasons.

The initial CVD event among our study patients may have occurred several years before the first-time MI. A 2-year adherence to statin therapy of 36.1 % for chronic coronary disease has been reported among elderly patients, and it is even less for primary prevention [52].

Concerns about side effects is associated with low adherence to drug therapy [53, 54]. Consequently, discontinuation of statins within 6 months after initiating therapy has been attributed to negative statin-related news reports in public media [55].

Discontinuation of previously initiated statin therapy due to side effects or negative public media reporting may have contributed considerably to the low number of patients receiving statins for secondary prevention. Follow-up of patients remains essential, even in patients with symptoms attributable to drug treatment, as most patients that are rechallenged with a statin are still able to tolerate statins long-term [56].

The usage of clinical guidelines in clinical practice is known to be incomplete [30, 57, 58], and the adoption of new treatment targets may be a slow process in general practice [35, 38, 39]. Patients’ understanding of the CV risk concept may also be an important barrier for preventive treatment [36].

Because we recruited patients of all ages, comparisons to population-based cohorts from restricted age segments should be made with caution. According to the “Irish Longitudinal Study on Ageing” [59], the proportion of patients undergoing statin treatment was 68.8 % for patients with known CVD, 57.4 % for diabetic patients, and 19.7 % when SCORE of risk was ≥5 %. Statin treatment in 49.1 % of our diabetic patients was possibly related to a twice yearly follow-up programme for diabetics. The modest rate of statin treatment in patients with prior CVD (34.5 %) may have been related to a lack of follow-up for CVD patients in our study area.

Women ≥70 years of age were less frequently on current statin treatment than men in the same age group or younger women possibly due to combinations of several causes. Female gender and age (>80 years) are independent risk factors for statin-associated muscle symptoms [12, 60]. A possible interaction between age and gender in relation to discontinuation of statin therapy should be researched further.

Awareness of personal CV risk profiles may be important for adherence to medication. In a recent survey conducted in women, underestimation of CV risk was common, with age as the most significant predictor [61]. In our cohort, only 3/70 patients with a SCORE ≥5 % for CV risk received statin treatment. Apparently, CV risk assessment by the SCORE chart was not implemented in clinical practice during the study period.

The reasons for the lower proportion of overall statin treatment among patients from primary care clinics with more permanent staff GPs (low short-term clinics) are not clear based on the study data. To provide more supporting evidence concerning the prescription preferences of GPs at different experience levels, a qualitative study approach could have been applied, but it was beyond the scope of our present study.

### Future aspects

The gap between CVD guidelines and clinical practice has been reported previously [62, 63], but the age- and gender-related aspects of this gap has not gained sufficient attention. To bridge this gap it is necessary to develop and implement procedures for case finding, recall, and follow-up of patients with established CVD [64, 65]. Such procedures should be integrated into electronic medical records as much as possible. Algorithms for CV risk assessment should be fully implemented in clinical practice, since single risk factor assessment could lead to false conclusions of CV risk, with potential overuse of drugs.

### Limitations

We enrolled MI patients admitted to the hospital; therefore, our results are not fully comparable to findings in community-based cohorts. Data on the time of the initiation of statin treatment, discontinuation of treatment, and side effects were not available. In most patients, angina pectoris, one of the components of the CVD diagnosis, was determined as a clinical diagnosis and not evaluated by coronary angiography. In medical practice, treatment decisions are often based on clinical risk evaluation, and invasive procedures are not always required or justified [66, 67]. In patients aged 40–65 years, the SCORE of CV risk [23] was determined from baseline data a posteriori. Due to a bidirectional relationship between statin therapy and the SCORE value, in patients achieving lower cholesterol levels after initiation of statins, the SCORE variable was not included in the regression model of determinants of statin treatment. Pre-treatment cholesterol levels were not available.

A post hoc calculation of sample sizes required to detect a difference in statin treatment with respect to primary care clinics’ use of short-term GPs, stratified by prior CVD, revealed a need for a larger sample size to reach a power of 0.8 at a significance level of 0.05. Thus, the study had insufficient power to detect a between-clinic difference in the use of statins after stratification by prior CVD.

We compared primary care clinics by the use of GPs working on short-term contracts vs. permanent staff GPs as a substitute for a measurement on the provider-patient level. Direct measurement at the level of care was not possible to obtain within the resources of this study.

Non-surviving patients due to out-of-hospital cardiac arrest were not included in the study, which limits the results to being applied to patients alive after a first-time MI.

## Conclusions

In patients with prior CVD we found considerable under-treatment with statins, especially among women and the elderly. In the entire study cohort, women ≥70 years of age had lower probability of statin treatment than men of the same age or younger women, after adjusting for diabetes and prior CVD. Overall, patients from clinics with predominantly permanent staff GPs received statin therapy less frequently than those with GPs on short-term contracts. Methodologies for case finding, recall, and follow-up of CVD patients need to be improved and implemented to achieve the goals for CVD prevention in clinical practice.

## Abbreviations

ACE, angiotensin-converting-enzyme; ACS, acute coronary syndrome; ARB, angiotensin-receptor blocker; CABG, coronary artery bypass grafting; CV, cardiovascular; CVD, cardiovascular disease; DDD, defined daily dose; GP, general practitioner; LDL, low density lipoprotein; MI, myocardial infarction; NAILED ACS, The Nurse-Based Age Independent Intervention to Limit Evolution of Disease After Acute Coronary Syndrome Risk Factor Trial; PAD, peripheral artery disease; PCI, percutaneous coronary intervention; RCT, randomised placebo-controlled trial; SCORE, systematic coronary risk intervention; TIA, transitory ischaemic attack.

## Additional file

Additional file 1: Estimated probability of statin treatment (95 % CI) in patients with prior cardiovascular disease by age. (JPG 19 kb)
