# Supplementary figures and images for: Low use of statins for secondary prevention in primary care: a survey in a northern Swedish population
Source: BMC Fam Pract. 2016 Aug 11;17:110. doi: 10.1186/s12875-016-0505-0 (PMC4982203; doi:10.1186/s12875-016-0505-0)

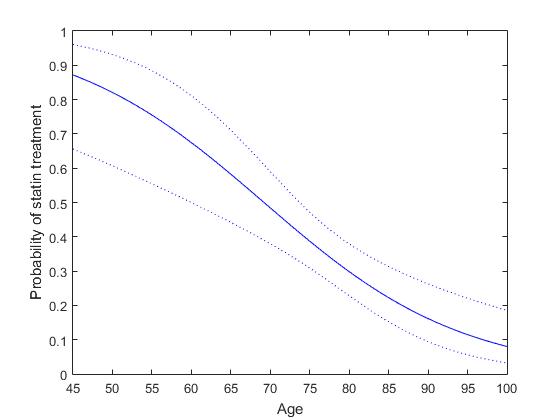

Supplement: Additional file 1: — Estimated probability of statin treatment (95 % CI) in patients with prior cardiovascular disease by age. (JPG 19 kb) [file 12875_2016_505_MOESM1_ESM.jpg]
